# Process evaluation of Samoa’s national salt reduction strategy (MASIMA): what interventions can be successfully replicated in lower-income countries?

**DOI:** 10.1186/s13012-018-0802-1

**Published:** 2018-08-06

**Authors:** Kathy Trieu, Jacqui Webster, Stephen Jan, Silvia Hope, Take Naseri, Merina Ieremia, Colin Bell, Wendy Snowdon, Marj Moodie

**Affiliations:** 1 The George Institute for Global Health, University of New South Wales, Sydney, Australia; 2 Sydney School of Public Health, Faculty of Medicine, The University of Sydney, Sydney, Australia; 3 Deakin Health Economics, Centre for Population Health Research, Faculty of Health, Deakin University, Geelong, Australia; 4 Global Obesity Centre, Faculty of Health, Deakin University, Geelong, Australia; 5 Ministry of Health Samoa, Apia, Samoa

**Keywords:** Sodium, Salt, Nutrition, Public health, Process evaluation, Hypertension, Cardiovascular disease

## Abstract

**Background:**

Evidence for recommended interventions to reduce population salt intake come from high-income countries, but it is unknown if these can be successfully replicated in low- and middle-income countries. This process evaluation investigated the reach, dose/adoption, fidelity, cost, and context of a national salt reduction program of interventions in Samoa.

**Methods:**

Monitoring and Action on Salt in Samoa (MASIMA) was a pre- and post-intervention study of a government-led strategy to lower population salt intake comprising awareness campaigns, community mobilization and policy and environmental changes. Data relating to the five process evaluation dimensions were collected from routinely collected data, a post-intervention survey and stakeholder interviews. Chi-squared tests assessed differences in quantitative survey responses among groups. Thematic analysis of qualitative interview responses was undertaken and triangulated with the quantitative data.

**Results:**

Awareness campaigns, school nutrition standards, and community mobilization interventions were implemented with moderate reach and fidelity. Higher than expected costs of campaigns and limited opportunity (one-off) to mobilize community leaders to disseminate salt reduction messages were key implementation challenges, which meant intervention dose was low. Environmental-level initiatives including engagement with the food industry to voluntary reduce salt in foods and the introduction of salt-related regulations were more challenging to implement within 18-months, particularly given the delay in the passing of the Food Act which provides for enforcement of regulations. Contextual factors that hindered the interventions’ mechanism of effect include the food culture, higher cost, and lower availability of healthy low-salt foods relative to unhealthy foods and salty taste preference.

**Conclusion:**

Although individual and community-based interventions helped increase awareness about the importance of salt reduction in Samoa, legislative backing was needed to alter the food environment to achieve population reduction in salt intake. It was not possible to engage the food industry to lower salt in foods through a voluntary approach in Samoa’s current context, although such initiatives were successful in some high-income countries. Future individual and environmental-level interventions to reduce salt intake need to address the contextual influences of food choices. In Samoa, this means salt reduction strategies need to ensure consuming lower salt is affordable, widely available, and perceived as flavorsome.

**Electronic supplementary material:**

The online version of this article (10.1186/s13012-018-0802-1) contains supplementary material, which is available to authorized users.

## Background

Excess salt consumption is strongly associated with raised blood pressure, a major risk factor for cardiovascular disease [1, 2]. In 2010, nearly one of every five premature cardiovascular-related deaths worldwide were attributed to salt intake above the recommended levels and most of these deaths occurred in low- and middle-income countries (LMICs) [3]. Evidence from a meta-analysis of 34 randomized trials demonstrated that reduced salt intake led to a decrease in blood pressure in hypertensive and normotensive individuals [1]. Furthermore, a recent global modeling analysis found that a government-supported strategy that reduced salt intake by 10% was considered cost-effective across 182 of 183 countries, with the best cost-effectiveness ratio estimated for lower-middle income countries [4]. Despite this, there is limited evidence of successful population programs to reduce salt intake in LMICs and most of the successful national programs are in high-income countries [5–8]. Therefore, most salt reduction initiative frameworks or guidance [9, 10] have been developed based on successful programs in high-income countries such as the UK [11], Finland [12], and USA [13], but it is unknown whether these types of initiatives can be successfully replicated in LMICs. Common interventions included in most successful strategies are government engagement with food industry to lower salt content of processed foods, public awareness campaigns, and healthy food procurement policies in public institutions (e.g., schools and hospitals) [5, 8, 10, 11].

In 2013, Monitoring and Action on Salt in Samoa (MASIMA) was initiated as part of a National Health and Medical Research Council (NHMRC), Global Alliance for Chronic Diseases Hypertension project to examine the effectiveness of salt reduction initiatives in the Pacific Islands [14]. Pacific Island Countries (PICs) comprise a group of 22 small island countries and territories which face high and rising burden from diet-related diseases; poor diet is a major risk factor for non-communicable diseases (NCDs) which account for 65–85% of deaths [15, 16]. Samoa has a population of about 196,000 and is made up of two main islands, Upolu and Savaii, with the capital city Apia located on Upolu. The MASIMA project implemented an 18-month, multi-faceted national salt reduction strategy and measured the effect through change in 24 h urinary salt excretion and salt-related knowledge, attitudes, and behavior (KAB) before and after the intervention. The outcome evaluation found that while there were significant improvements in knowledge and self-reported behavior (intermediate outcomes), there were no changes in mean salt intake (7.3 g/d in 2013 vs 7.5 g/d in 2015; *p* = 0.588) [17]. These mixed findings highlight the importance of a process evaluation to aid interpretation of the results and improve future implementation of interventions.

Given the complexity of most public health interventions and the need to understand their impact in real-world settings, there is increasing recognition of the importance of evaluating interventions beyond their efficacy [18]. An understanding of the process alongside intervention efficacy, captures essential information about why an intervention did or did not achieve its intended effects within its context and how implementation can be optimized in future interventions [19]. Despite its importance, there are few process evaluations of programs to reduce population salt intake and none have been undertaken in a lower-middle income setting like Samoa or in a national strategy implemented by the government. The purpose of this study was to conduct a process evaluation to investigate the reach, dose/adoption, fidelity, cost, and context of MASIMA—Samoa’s national, government-led salt reduction strategy.

## Methods

### Interventions

Full methodological details of the MASIMA salt reduction outcome evaluation study have been previously published [17]. Based on the baseline salt intake and KAB survey in 2013, the Ministry of Health Government of Samoa (MoH) implemented an 18-month multi-faceted salt reduction strategy from March 2014 to September 2015. Two MoH project officers were recruited to the Strategic Planning Policy and Research Division to lead the salt reduction intervention and evaluation with support from other divisions in MoH, other Government Ministries, the World Health Organization (WHO), and The George Institute for Global Health.

**Fig. 1:**
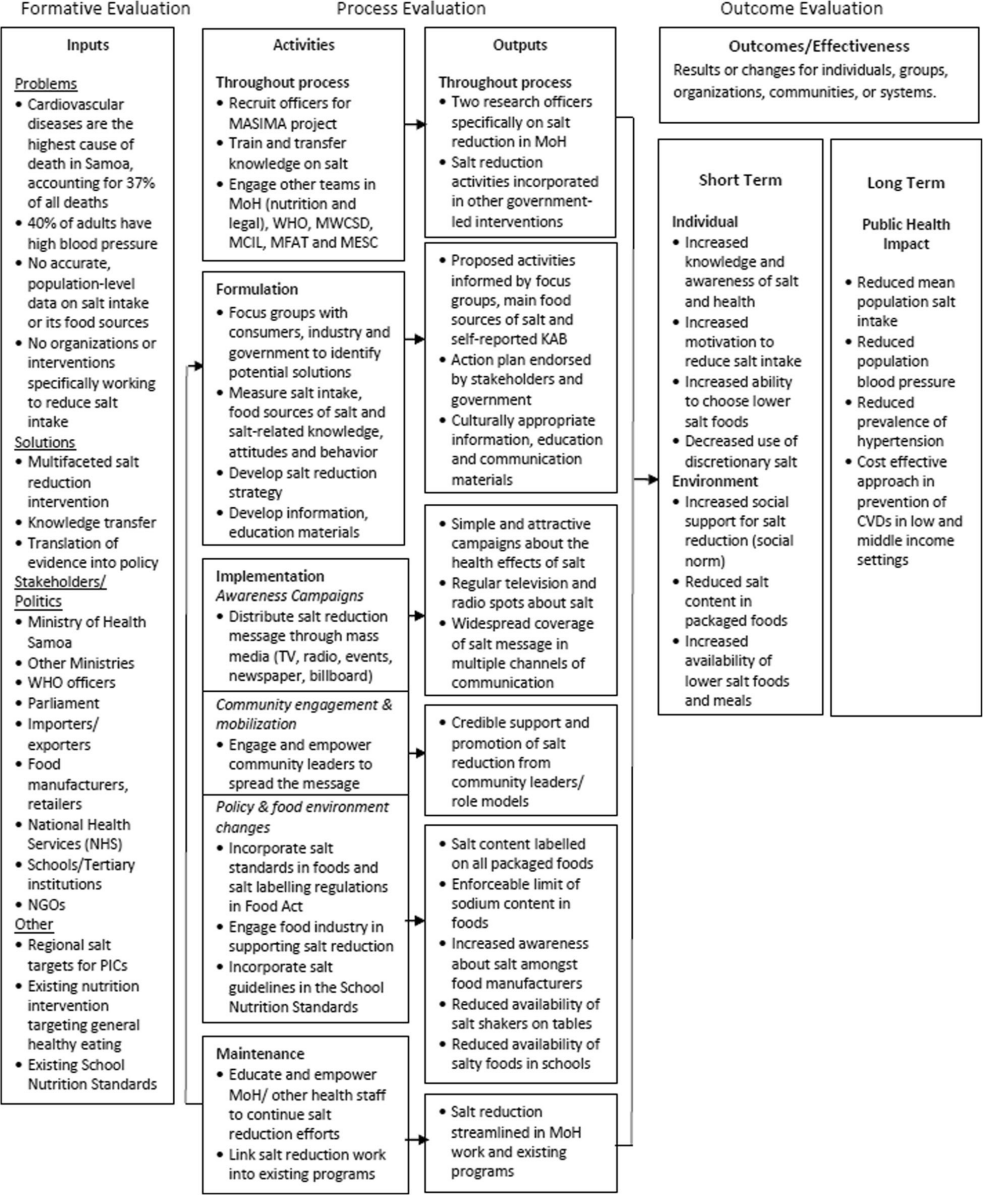
Logic model for Samoa salt reduction strategy adapted from the Center for Training and Research Translation evaluation framework

The MASIMA salt reduction strategy consisted of three strategic objectives that aligned with the three levels of factors that influence food choices as outlined by Story et al.’s ecological framework: individual, social environment, and physical or macro-level environment [20]. The selection of interventions was based on evidence of previously successful interventions that formed the WHO’s three main pillars of salt reduction (product reformulation, consumers, and the environment) [9], and adapted to the sources of sodium in Samoa and findings from focus groups with government, non-governmental organizations, civil societies, restaurants, caterers, food manufacturers, and importers [21]. A logic model (Fig. 1) outlines the causal pathways about how each of the interventions works and interacts to produce the intended intermediate and long-term outcomes. To influence the public’s salt reduction behaviors, the first strategic objective aimed to achieve widespread awareness about the adverse health effects of eating too much salt and to encourage salt reduction through mass media. This provided individuals with the understanding and motivation to change their salt consumption behaviors as this was low, according to the baseline KAB survey. The second objective was to engage and empower influential community leaders (village mayors, church leaders, educators) in providing credible support and advice for salt reduction. This aimed to influence the social environment as interactions with family, friends, and community leaders impact food choices through role modeling, social support, and social norms. The third objective aimed to create policies and food environments that support low salt consumption through three key activities: (a) incorporation of salt-related standards into the Food Act (now known as the Food Safety and Quality Regulations 2017 in accordance with the 2015 Food Act); (b) integration of salt guidelines into the mandatory school nutrition standards; and (c) engagement with the food industry (manufacturers, restaurants, caterers) to support salt reduction through voluntary commitments to produce or import lower-salt foods. Together, these aimed to influence the physical and macro-level environmental factors through increasing the availability and ease of eating lower salt foods and meals.

### Theoretical framework for process evaluation

The UK MRC guidance on process evaluation of complex interventions was followed [18]. This involves understanding the implementation of interventions, context in which they were delivered, and the mechanisms of impact. In conjunction, Steckler and Linnan’s framework was used to comprehensively examine the level of implementation in terms of reach, dose, and fidelity (Table 1) [22]. Due to the multilevel nature of some interventions, an organizational level indicator, adoption, was also included from the RE-AIM framework [19]. The cost of interventions was also documented to indicate the level of implementation and transferability of interventions, particularly to other LMICs with limiting funding [23]. Table 1 describes the process evaluation dimensions.

**Table 1:** Process evaluation dimensions and definitions

Dimension	Definition
Reach	The proportion of the target audience that encounters or participates in the intervention and its representativeness [19, 22]
Dose	The quantity of the intervention that participants received (dose received) or the quantity of the intervention delivered (dose delivered) [18, 22]
Adoption	the number of settings or institutions that adopt a program or policy [19]
Fidelity	The quality of what is delivered and the extent to which the intervention is delivered as planned [18, 22]
Context	The contextual factors (facilitators or barriers) that influence implementation and the mechanisms of impact or outcome [18]
Cost	The cost of the interventions from a government perspective

### Data collection

**Fig. 2:**
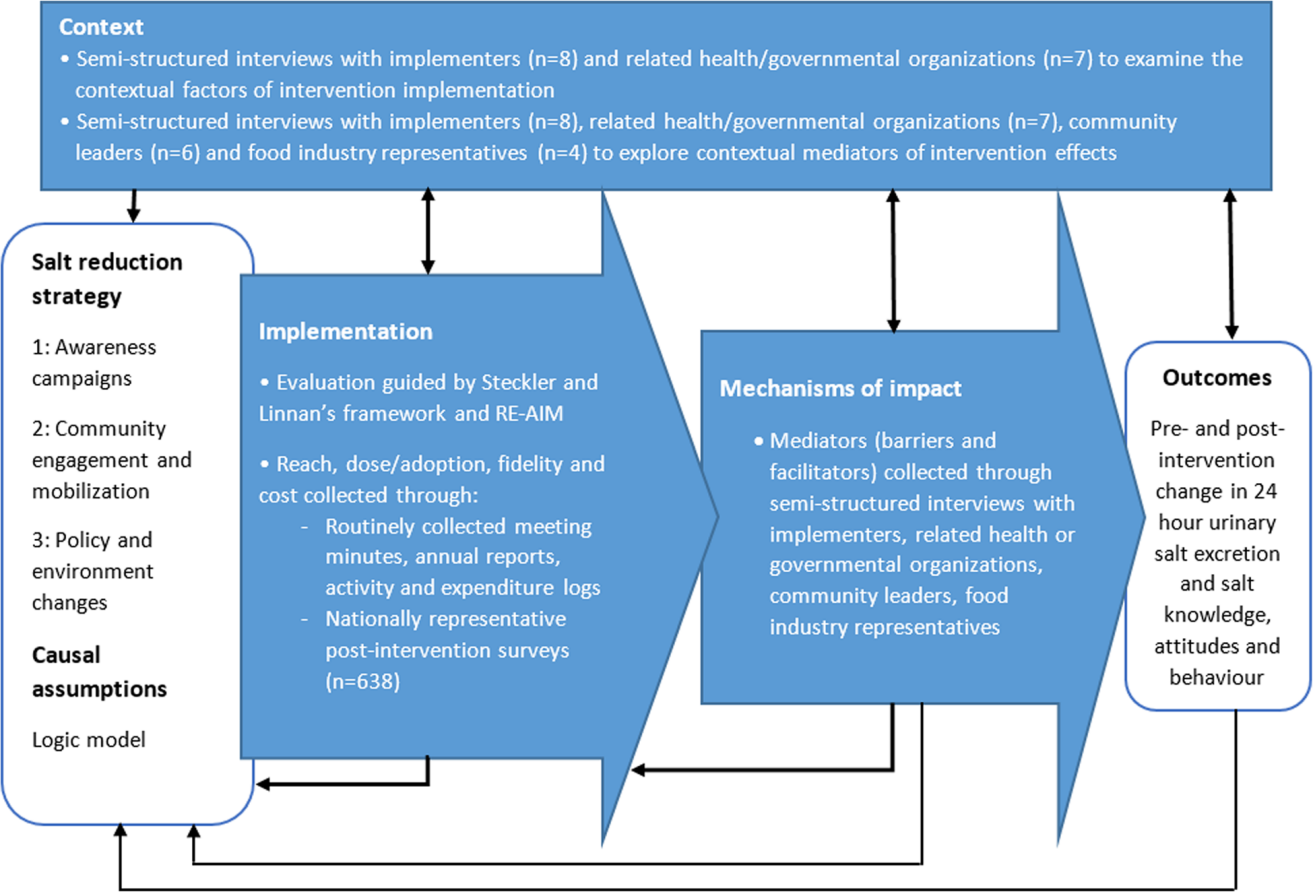
Data collection methods and frameworks used for the process evaluation adapted from the UK MRC guidance

A mixed methods approach was utilized comprising three main sources: data routinely collected during the intervention, national post-intervention survey responses, and semi-structured interview responses (Fig. 2). Routinely collected data from activity logs, meeting minutes, and annual reports provided detailed information on the intervention activities, personnel involved, duration, frequency, resources used, and cost. Intervention cost data were collated by Deakin University based on project records of resources used. A government perspective was adopted meaning that all costs expended by any government ministry in the delivery of the interventions were included. This perspective was chosen because the multi-faceted intervention not only involved the MoH but also other government ministries such as Ministry of Education, Sport and Culture (MSEC) and Ministry of Women, Community and Social Development (MWCSD). All costs were expressed in Samoan Tala (WST) for the year 2014 (1 Tala in 2014 = 1.04 Tala in 2017 = 0.53 Australian dollar in 2017) [24].

Process evaluation questions related to the salt reduction campaign were added to the post-intervention survey. Using multi-stage cluster sampling, 780 randomly selected participants aged 18–64 years, in urban (Apia Urban Area) and rural regions (North West Upolu, Rest of Upolu and Savaii) in Samoa were invited to participate in the post-intervention survey. Details about the post-intervention survey methodology have been previously published [17]. The question ‘Have you heard about or seen any promotion regarding the Slash the Salt campaign?’ was included; if the participant answered ‘yes,’ they were asked ‘how.’

A project investigator (JW) undertook 25 semi-structured interviews in April 2016, as she had good knowledge of the project action plan but was not involved in the implementation of interventions, and she was the most culturally appropriate person to investigate the contextual influences until data saturation was reached. The interview questionnaires were designed to obtain an insight into intervention fidelity and how context may have affected implementation and outcomes. Semi-structured interviews were held with eight MoH staff who had varying levels of involvement with the project, seven members from related health or government organizations, six community leaders, and four food industry representatives (Table 2). Interview participants were purposely chosen to obtain views from a wide range of stakeholders, with varying levels of involvement in the intervention and representing different types of national organizations and community groups. Interview participants provided written consent for participating and having the interviews recorded.

**Table 2:** Semi-structured interview respondents

Group	Interviews	Detail
Ministry of Health Samoa	8	Two salt research officers and six people (Chief Executive Officer of MoH, two Assistant Chief Executive Officers of Divisions, and three principal officers) who had varying levels of involvement in the intervention.
Related health or governmental organization	7	Two staff from WHO, one from MWCSD, two from National Health Services, one from MESC, and one from Samoan Bureau of Statistics
Community leaders	6	Two educators, two television and/or radio hosts, and two youth leaders
Food industry	4	Two local food manufacturers, one catering company, and one retailer

*MoH* Ministry of Health, Samoa, *WHO* World Health Organization, *MWCSD* Ministry of Women, Community and Social Development, *MESC* Ministry of Education, Sport and Culture

### Data analysis

Routinely collected data on the intervention details and costs were compiled and organized in an Excel database by the five interventions components: awareness campaigns, community engagement and mobilization, and the three policy and environment activities (salt-related regulations in the Food Act, salt guidelines in school nutrition standards, and food industry engagement). Data from all participants who completed the questionnaire post-intervention and had no missing information were included. Chi-squared tests were conducted to assess differences in reach and dose between different demographic and clinical groups (age, gender, locality, education, employment, and hypertension diagnosis). Statistical analyses were conducted using STATA IC version 13.0 (StataCorp LP, College Station, Texas, USA).

Semi-structured interview responses were transcribed and coded by an independent student not involved in the study (PG) using NVivo (version 10; QSR International, Doncaster, VIC, Australia). The lead author (KT) also separately coded the interview responses in NVivo, and any differences were resolved through discussion. A thematic analysis of the interview responses was undertaken based on the key process evaluation dimensions of each of the five strategy components. Quantitative and qualitative data were triangulated where relevant [25]. For example, when the high cost of campaigns was identified as an emerging theme in interview responses about the barriers of implementation, this was triangulated with quantitative data on the cost of each intervention.

## Results

For each dimension, where available, we present the quantitative findings for each of the three main intervention components (public awareness campaign, community engagement and mobilization, and policy and environmental changes) and qualitative findings which provides context and support for the quantitative results (Tables 3 and 4). The interview responses have been summarized in the results and specific quotations can be found in Table 5, Additional files 1 and 2.

**Table 3:** Summary of reach, dose/adoption, fidelity, context, and cost by intervention component

Domains	Public awareness campaigns	Community mobilization and engagement	Policy and environmental changes		
			Salt-related regulations	School nutrition standards	Food industry engagement
Reach	High	Moderate	Nil^a^	Low	n/a
Dose/adoption	Low	Low	Nil^a^	Moderate	Low
Fidelity	High	Moderate	Low	High	Low
Context influencing implementation	Moderate	Favorable	Unfavorable	Moderate^b^	Unfavorable
Context influencing intervention effect	Unfavorable	Moderate	Nil^a^	Unfavorable	Unfavorable
Cost	High	Moderate	Low	Low	Low

^a^Salt-related regulations were not been passed within the duration of the project so assessment against these domains were not undertaken

^b^No barriers or facilitators of implementing salt standards into the school nutrition standards were identified

**Table 4:** Reach and dose of the awareness campaigns by subgroups (*n* = 638)

	Heard/saw campaign (%)	Mean sources of exposure (sd)	Stickers (%)	Radio (%)	Television (%)	Community event (%)	Printed materials (%)	Billboards (%)	Family/friends (%)
Gender									
Female	75.97	1.40 (1.19)	4.42	30.66	67.68	11.05	17.13	4.42	4.42
Male	73.19	1.30 (1.15)	1.45	29.71	63.41	9.78	16.67	5.80	3.26
*p* value	0.423	0.30	*0.03*	0.80	0.26	0.61	0.88	0.43	0.46
Age, years									
18–44	72.73	1.38 (1.23)	4.04	28.79	65.66	10.10	19.19	6.31	4.04
45–64	78.10	1.31 (1.09)	1.65	32.64	66.12	11.16	13.22	2.89	3.72
*p* value	0.130	0.48	0.09	0.30	0.91	0.673	*0.05*	0.06	0.84
Locality									
Rural	72.67	1.32 (1.19)	3.49	30.62	62.79	9.88	15.89	5.04	4.07
Urban	83.61	1.52 (1.13)	1.64	28.69	78.69	13.11	21.31	4.92	3.28
*p* value	*0.012*	0.09	0.29	0.68	*< 0.01*	0.30	0.15	0.96	0.69
Employment									
Employed	76.79	1.51 (1.28)	3.80	33.76	63.71	13.08	24.05	8.02	5.06
Unemployed/not economically active	73.57	1.26 (1.10)	2.74	28.18	67.08	8.98	12.72	3.24	3.24
*p* value	0.365	*< 0.01*	0.46	0.14	0.39	0.10	*< 0.01*	*< 0.01*	0.25
Highest level of education completed									
Primary school or less	68.63	1.19 (1.08)	0.78	28.63	56.08	9.80	14.90	3.53	5.10
High school or above	78.85	1.47 (1.23)	4.70	31.33	72.32	10.97	18.28	6.01	3.13
*p* value	*< 0.01*	*< 0.01*	*< 0.01*	0.47	*< 0.01*	0.64	0.27	0.16	0.21
History of hypertension									
No	73.93	1.35 (1.15)	3.15	31.46	64.49	9.66	16.63	5.84	4.72
Yes	76.68	1.36 (1.19)	3.11	27.46	68.91	12.44	17.62	3.11	2.07
*p* value	0.462	0.90	0.98	0.31	0.28	0.29	0.76	0.15	0.11

*P* value <0.05 was considered significant, as shown in italics

**Table 5:** Interview quotes of contextual barriers and facilitators

Theme	Interview respondent	Quote
Contextual factors affecting implementation	Related health or government organization representative	“…they spoke to the women committee members, and that’s how they got the discussion back to the village. …In Samoa it’s very appropriate to do it like that. You cannot do it otherwise. …the women committee members they are always the ones that, for instance, the president is the wife of the high chief of the mayor, the secretary of the committee is the wife of the church minister.”
	Ministry of Health representative	“For the Food Act, we were hoping for that to back us up so that we can integrate the salt targets… I think it’s just unfortunate now the project is towards its end and now we have the Food Act. …As I have said, we have been waiting for this Food Act because we are also looking at labelling, an area we really need to push.”
Contextual factors affecting the outcome of awareness campaigns	Community leader	“People love to eat here, and they love to eat salt.…Everything is about food here. … Everyone here lives for today. Yeah we want a good future but you are really living for the moment because not everyone has the food on their plate the next day. …So you are dealing with a huge cultural issue.”
	Related health or government organization representative	“It’s like the noodles. …Yeah because it’s affordable, it’s easy to get. …Even though you know the awareness says it’s not good for health, but what else are we supposed to eat if we do not have enough money.”
	Community leader	“…And it’s money in your pocket and that’s what people actually care about, not your health because you do not know what’s inside those packets, you do not know what those numbers [salt content on nutrition label] mean or anything. There’s a big lack of education about what’s right for you”
	Related health or government organization representative	“There’s a lot of fitness programs. A lot of people are conscious of what they eat now. … So I think there’s a change from ten years ago. We love to feast whenever we have a chance. But now even when we order our food for events, we are really conscious, we really want to have healthier options.”
Contextual factors affecting the outcome of community mobilization	Related health or government organization representative	“So part of the community awareness, the villagers were told to think of a project… And what do you know? Salt. …one of the villagers in Savaii said the reason why we want to do Masima is because we went to the community engagement that was done by the Ministry of Women. So I think for the PEN FA’A SAMOA there was seven sub-villages and I think you have four [doing salt reduction]… So that’s probably one of the things you can measure. Output of the MASIMA project. Also that it’s been continued by this PEN FA’A SAMOA.”
	Food industry representative	“They [Ministry of Health officers] came down and spoke with me and I said my problem right now is salt sells, so does sugar. I really prefer Samoans to eat natural foods and to always eat what they traditionally ate. But unfortunately I am competing against massive advertising campaign from the producers overseas here so I have to produce what the Samoans are eating. …If I do not react to the market demand and I try to dictate to the market, I will lose.”
	Ministry of Health representative	“… the salt progress report was presented and in this committee it’s all of the prime minister, cabinet, all ministries, their CEO and their management. …And the minister of finance said ‘you need to submit a paper and need to raise the taxes on salt’, which was a really good indication coming from the minister of finance”

### Reach

Of 780 participants randomly selected for the post-intervention survey, 134 did not consent, 3 had missing demographic data, and 5 did not complete the process evaluation questions, leaving 638 participants eligible for analysis (82% response rate). Overall, 75% of the Samoan adult population reported that they had heard or seen the ‘Slash the Salt’ intervention. The reach ranged from 64% in the most rural region (Savaii) to 84% in urban region Apia. The leading source of exposure was television (66%) followed by radio (30%), posters/leaflets (17%), community events (11%), and billboards (5%) (Table 4). Urban residents were significantly more likely to recall hearing or seeing the intervention compared to rural residents (84% vs 73%, *p* = 0.01). Respondents with higher education were more likely to recall exposure to the intervention (79% in high school completers or above vs 69% in those who completed primary school or less, *p* < 0.01). There were no associations between age, sex, employment, or reported hypertension and exposure to the intervention (*p* > 0.13 for each).

Approximately 590 community leaders were educated and mobilized to promote messages about salt reduction at four separate events. This consisted of 191 “Sui Tama’itai” (village women representatives or mayors that each represent 1 village out of 330 villages in Samoa), 144 faith-based or youth leaders, 205 teachers, and 50 mothers. Seventy-four percent (77 of 104) of village women representatives in Savaii (rural island) and 50% (114 of 226) of village women representatives in Upolu Island attended the salt awareness talk organized by the MWSCD. As part of the environmental initiative to incorporate salt guidelines into the mandatory school nutrition standards, MoH nutritionists visited 25% (52 of 209) of all schools nationwide to raise awareness about the types of salty foods that should not be sold and the importance of eating low-salt foods to school canteen operators, teachers, and parents. The reach of the salt standards were not measured, as the regulations were not passed during the intervention period.

### Dose or adoption

Overall, the dose delivered or received by the target audience or adoption of interventions by organizations was low*.* Based on the post-intervention survey, the mean and median number of sources of the salt reduction intervention recalled by participants were 1.36 and 1 respectively. Some 37% of respondents identified hearing or seeing one source of exposure, 21% identified two sources, and 11% identified three sources of exposure. The mean number of sources of exposure identified was significantly different among respondents of different education levels (1.47 in high school completers or above vs 1.19 in non-high school completers, *p* < 0.01) and employment status (1.51 in employed vs 1.26 in unemployed or not economically active participants, *p* < 0.01). There was no difference in mean exposure among different groups of age, sex, locality or history of hypertension (*p* > 0.09 for each) (Table 4). Similarly, the dose of the community mobilization interventions was low as all awareness talks were delivered once and each ranged from about 15 to 30 min.

Approximately 40% of 166 government schools adopted and complied with the nutrition standards (which included salt standards) in the 2014–15 financial year. As part of efforts to engage the food industry to promote a lower salt environment, 17% (26) of 151 food outlets in Apia (8 restaurants, 16 food stalls in Savalalo Fleamarket and 2 food manufacturers) agreed to display posters and leaflets about salt reduction and remove salt shakers from tables during World Salt Awareness Week in 2015. The adoption of salt-labelling and salt content regulations by food manufacturers and importers was not measured as the regulations were not passed during the intervention period.

### Fidelity

Fidelity was determined by comparing the planned activities outlined in the strategic action plan with delivered activities reported in annual reports, meeting minutes, and the costing documentation. This comparison showed that all public awareness activities and activities to incorporate salt guidelines into the nutrition standards in schools were implemented as planned. The fidelity of community mobilization activities was moderate, with some tasks not implemented as extensively as planned. For example, salt reduction messages were integrated into one existing community health outreach program as opposed to all, as planned. Similarly, the fidelity of interventions to engage food industry was low, because education and training sessions to food caterers about using less salt was not undertaken. The fidelity of the strategy to incorporate salt-related regulations into the Food Act was also low as mandatory labelling of the salt content and salt standards for packaged foods were not introduced as planned.

### Contextual factors affecting implementation

Contextual factors (barriers and facilitators) of implementation of the intervention were collated from the 25 semi-structured interview responses (Additional file 1). Five respondents expressed that a barrier to the implementation of the overall intervention was a lack of staff undertaking a large project. For most of the 18-month intervention time, there were two officers working solely on the salt reduction project. More specifically, the only barrier to implementation of the awareness campaigns identified by six respondents was the higher than expected costs, which limited the dose or amount of campaign materials that could be produced. Similarly, a barrier of implementation for the community mobilization intervention identified by one respondent was that the education sessions to empower the community leaders were only one-off sessions. In contrast, nine respondents identified that a facilitator of implementation was being able to utilize an established mechanism organized by the MWCSD, where village women representatives had a role to liaise with government agencies and relay the messages back to their village. Some added that mobilizing the village women representatives in particular was advantageous as women were often responsible for the cooking in Samoa, they are often influential people and considered role models.

Lastly, the main barrier of implementation of the policy and environmental interventions was the delay in the passing of the overarching 2015 Food Act, which sets provisions for food regulations and enforcement mechanisms. Three interviewees explained that the Food Act was not passed until June 2015 (3 months prior to the end of the intervention period). This delayed the proposals to incorporate salt-labelling and salt standards for foods into the regulations within the project timeframe. Similarly, the delay in the Food Act may also explain why the intervention to engage food industry in voluntary salt reduction was not delivered as extensively as planned. Three interview respondents said that it was important to wait for the Food Act to be passed before asking food manufacturers (particularly local companies) and restaurants to voluntarily commit to lowering the salt content of their foods. The reasons for this were that the Food Act was needed to substantiate the MoH’s request for food manufacturers to lower the salt content of foods and so that the requirements would be applied equally to all food manufacturers, including global manufacturers, not only the local food companies that were more easily contactable.

### Contextual factors affecting the outcome or mechanism of effect

The semi-structured interview responses also revealed several contextual factors that potentially influenced the interventions’ effects and were categorized by each of the three intervention target groups: the general public, community leaders, and food industry organizations (Additional file 2).

#### Contextual factors affecting the outcome of awareness campaigns

The effects of the public awareness campaigns were hindered by Samoa’s food culture and taste preferences, the cost and availability of healthy/lower-salt food compared with unhealthy foods and limited nutrition knowledge. Thirteen respondents explained that Samoans believe that food without salt is tasteless; it is part of their cultural food practices to add salt during the preparation of foods and at the table and to feast on as much tasty/salty food as desired because they prefer to live for today (Table 5). The second common barrier identified by nine respondents was that most people choose unhealthy food because healthy foods are perceived as more expensive, less convenient and unavailable, even if they know salt is bad for their health. Lastly, 11 respondents indicated that health literacy and nutrition knowledge in Samoa were low. The implications of this varied, with some respondents suggesting people still do not have the knowledge about how to reduce salt intake or to identify foods high in salt, whilst others suggested this meant awareness messages needed to be simple to be understood. This can provide context for the subgroup findings showing people with lower education were less likely to recall the salt reduction messages, because they are unlikely to recall hearing the message if they did not understand it. In contrast, 10 respondents identified a contextual facilitator that may have strengthened the intervention effects is the growing trend towards healthier behaviors, suggesting that the project was implemented at an opportune time.

#### Contextual factors affecting the outcome of community mobilization

The pathways to impact population salt intake through mobilizing community leaders to provide credible support for the salt reduction messages were potentially influenced by two barriers recognized by two interviewees. One stated that the village women representatives could only relay the salt reduction information that they had been provided in the one-off session, so it limited their scope to explain the issue or answer questions. The other suggested that communities had been bombarded with messages from their village women representatives for years, so something different was needed to get the salt reduction message across. In contrast, four respondents suggested that a parallel project called PEN FA’A SAMOA (based on WHO’s Package of Essential Non-communicable disease interventions in primary health care) strengthened the salt reduction message. Four of seven villages selected salt reduction as their PEN FA’A SAMOA project topic, having heard about it through the village women representatives, which meant the salt reduction message was continued.

#### Contextual factors affecting the outcome of policy and environmental interventions

The pathways of impact of the policy and environmental initiatives were also negatively affected by contextual factors. Three out of four food industry representatives expressed that despite the government’s efforts to encourage them to voluntarily produce or sell lower salt foods, their concern was that Samoans do not prefer lower salt foods, so they are less likely to sell and are less profitable for their business (Table 5). One added that mandatory, rather than voluntary, salt content standards for foods were preferred, so that there was an even playing field among food companies (including global companies). Three non-food industry respondents also thought that saltier foods increased sales and industry are mostly concerned with their profits, so they are unlikely to voluntarily lower salt content in foods. Another barrier advised by one industry respondent was that salt is used as an inexpensive preservative in Samoa where the climate is hot and refrigeration is uncommon. Lastly, three respondents identified that the effect of mandatory school nutrition (including salt) standards was likely to be reduced by the presence of street vendors selling non-compliant, unhealthy foods outside schools.

One potential facilitator of the adoption of nutrition standards and promotion of salt reduction messages in schools was the overall positive attitude towards the need to improve children’s nutrition and nutrition knowledge, as expressed by six respondents. Four respondents identified an unexpected result of the salt project and proposals to the Cabinet Committee for salt-related regulations, was that it contributed to the political readiness for mandatory action as demonstrated when the Minister of Finance proposed that a tax on high salt foods should be considered.

### Intervention costs

Based on the routinely collected cost data, the total salt reduction strategy costed WST 271,711 over 18 months (Table 6). Personnel costs for two research officers’ time across the project made up 39% of the total project costs. The public awareness campaigns were the most expensive intervention costing WST 94,665 or 35% of the total cost. This supports interviewee responses which identified campaign costs as a barrier to implementation. This is followed by the community mobilization intervention (21% of costs) and of this, the most expensive activity were the awareness talks with village women representatives as they were provided with monthly allowances and travel costs and catering. The three policy and environmental initiatives made up 5% of costs and mainly consisted of the food inspectors’ time engaging food outlets to remove salt shakers from tables, the provision of campaign material to support salt reduction and MoH nutritionists’ time in encouraging the adoption of the new salt standards in schools. Given that 75% of the adult population (18–64 years) heard or saw the salt reduction message, this meant the overall intervention costed WST 3.7 per adult reached.

**Table 6:** Intervention costs (Samoan Tala) from a government perspective

Intervention component	Total costs		Personnel costs		Other costs (print material, travel, venue, etc)	
	WST	%	WST	%	WST	%
Research officers across all intervention components	105,439	39	105,439	65	0	0
Awareness campaigns	94,665	35	28,681	18	65,985	61
WSAW 2015	71,127		23,593		47,534	
WSAW 2014	11,700		143		11,558	
Health promotion officers	3760		3760		0	
Other (TV, booths, materials)	8078		1185		6893	
Community engagement and mobilization	56,728	21	23,297	14	33,431	31
Village women representatives meeting	19,317		11,597		7719	
Other community engagement activities	22,630		11,700		10,930	
IEC materials and other	14,782		0		14,782	
Salt-related regulations	2425	1	0	0	2425	2
School nutrition standards	5513	2	5,76	3	437	0.4
Engagement with food industry	6941	2	665	0.4	6277	6
Total	271,711	100	163,157	100	108,554	100

*WST* Samoan Tala, *%* percentage, *WSAW* World Salt Awareness Week, *IEC* Information, Education and Communication materials

## Discussion

The MASIMA study process evaluation provides detailed information on intervention implementation and context to better understand why improved intermediate outcomes (salt knowledge and self-reported salt-lowering behaviors) were achieved, but there was no change in mean population salt intake in Samoa. This understanding elucidates recommendations for future strategies needed in Samoa and other similar LMICs, to achieve reduced salt consumption. Overall, the reach of the salt reduction strategy was high, fidelity was moderate, costs were low, and the intervention dose or adoption was low (Table 3). The public awareness campaigns were implemented with the highest reach, dose, and fidelity, followed by the school nutrition standards and the community mobilization intervention. In contrast, the food industry engagement intervention and the salt regulations were largely not implemented as planned during the intervention period, partly due to an unforeseen delay in the passing of the 2015 Food Act. From the perspective of Story et al.’s ecological framework of the multi-level influences on eating behavior [20], this meant the salt reduction interventions that were moderately implemented only targeted factors at the individual and the social environment level, whereas the physical or macro-level environmental factors were not addressed. Lastly, there were several contextual factors affecting implementation and outcomes, suggesting the replication of some successful interventions in high-income countries into lower middle countries like Samoa proved challenging.

The public awareness campaigns and community mobilization interventions, which were implemented with greater reach and fidelity, are likely to have contributed to the improved intermediate outcomes; a 9% increase in the population being aware of the adverse health effects of salt, 16% reduction in the population ‘always or often’ adding salt during eating, and 21% increase in self-reported behaviors to lower salt intake [17]. The awareness campaigns achieved widespread reach of 75% as recommended by the Centre for Disease Control for effective mass media communication [26] and equal reach among different age, gender, and employment groups (Table 4). Although costly (also found in other studies [4, 11, 27, 28]), the awareness campaigns were more feasible to implement from a government perspective. This is consistent with a global review, which found 71 of 75 countries have implemented an education or awareness campaign as part of their national salt reduction strategy [5]. Although increased levels of motivation and awareness about the adverse health effects of excess salt intake were achieved, this alone is unlikely to alter usual salt intake [29].

Interviews revealed that the general population still lacked knowledge about how to lower salt intake and identify foods that are high in salt content, which may explain post-intervention findings of no changes in consumers ‘always or often’ adding salt during cooking or avoiding intake of processed foods [17]. The next phase of the campaigns and behavior-change communications should focus on improving people’s capability to lower salt intake [30]. The UK’s successful salt reduction campaign consisted of four phases, transitioning from raising awareness about salt to highlighting foods with hidden salt and providing practical solutions for lowering salt intake [11]. The use of multiple channels of mass media should be continued however additional efforts are needed to ensure the message can be understood by people of lower education levels and delivered to people in rural areas, given there was significantly less reach in these groups. Future interventions should focus on increasing the dose delivered. Many interviewees regarded the mobilization of village women representatives as a good opportunity to reach rural villages but the downfall was related to the low dose. Future strategies should continue to educate these community leaders through ongoing sessions and in creative ways, such as cooking demonstrations using alternatives to salt and shop visits to teach community members how to interpret the salt content on packaged food labels [29, 31]. Where possible, initiatives should also link with other existing government initiatives (e.g., PEN FA’A SAMOA) to re-inforce the message.

Despite efforts to educate consumers about the importance of salt reduction for better health, there were several contextual influences of food choice that superseded health motivations. These included the taste preference for salty flavor, food culture, price, and availability. Other studies in Samoa and other PICs also identified that the strong cultural importance of feasting and people’s perception that salty food is tasty were barriers of healthy food consumption [32–35]. Although food prices are a determinant of food choices globally [36], in lower income countries like Samoa, cheap unhealthy foods were the only food options that some could afford [33]. Therefore, future behavior-change approaches to lower salt intake in Samoa must address these factors by educating consumers about using inexpensive and widely available alternatives to replace the taste of salt such as herbs, spices, and lemon juice during cooking or eating.

Processed foods and meals that already contain salt added by manufacturers or caterers are also a major source of salt in the Samoan diet. Therefore, policies and environmental-level interventions are crucial. The MASIMA initiatives to engage food manufacturers to voluntarily produce or import lower salt foods and introduce mandatory salt labelling and salt-content standards for foods were more challenging to implement in a limited time and faced more contextual barriers. Although the school nutrition standards were moderately implemented with about a 40% compliance rate in government schools, this change alone was unlikely to influence adults’ salt intake (the target population). Without environmental changes, even with improved individual awareness, it is difficult for individuals to sustain lower salt food consumption as healthy (low salt) foods are perceived to be more costly, less available, less flavorsome, and inconvenient to prepare compared to unhealthy foods [37]. This is a likely explanation for why there were no changes in mean salt intake in Samoa after 18 months of interventions, despite improvements in knowledge and self-reported behaviors. This is consistent with a review of solely behavior-change interventions, which found 18 of 19 studies showed significant improvements in self-reported behaviors related to salt, but only two of six demonstrated significant reductions in 24 h urinary salt excretion [29]. This emphasizes the importance of continued long-term efforts to implement policies and environmental interventions that enable the population to more easily consume lower salt, through making lower-salt processed food and meal options more affordable, widely available, and flavorsome.

Legislative policies to support lower salt intake in Samoa are more feasible now, as the MASIMA salt project has increased political readiness, and the 2015 Food Act has been passed [38]. Furthermore, since health is not a strong focus or determinant of food choice in Samoa, different incentives or regulation are needed. As food prices are a strong determinant of food choice, fiscal policies such as the taxation of salty foods in combination with subsidies for healthy foods (e.g., fruit and vegetables) would likely be effective in improving food choices [27, 39]. It is also likely to be feasible given the Minister of Finance in Samoa himself suggested taxation, and three countries currently have taxation of salty foods globally [5].

In Samoa’s context, government engagement with food manufacturers to voluntarily lower salt content in foods proved to be less feasible, despite success in several high-income countries [40]. Food industries were reluctant to voluntarily decrease salt in their products because they share the perception that food with less salt is less tasty, and therefore less likely to sell. The success of the voluntary approach also relies on the notion that all food companies pull their weight in the salt reduction efforts; however, in Samoa, local food manufacturers felt that their global competitors were not engaged to do their part [41]. Local food companies indicated preference for mandatory policies as they felt they were disadvantaged under the voluntary system, as they would be targeted by the government over global food manufacturers, which are harder to reach and engage. The parallel project in Fiji, where interventions were implemented by researchers, identified similar challenges. Food manufacturers suggested salt reduction would not be a priority for them unless it was mandatory [28]. Mandatory salt reduction policies that create an even playing field for local and international food manufacturers are likely to be more effective and feasible than voluntary engagement in countries similar to Samoa and Fiji [38].

Documentation of intervention costs is useful for understanding the level of implementation and the potential for replication particularly in lower income countries. Limited studies that have collected costs during real-world implementation of salt reduction interventions [28]. Most studies previously estimated costs through expert opinion or the application of standard costs of interventions, for example, using the WHO NCD Costing Tool [42]. Overall, the total cost of the 18-month national salt reduction program from a governmental perspective was relatively low (WST 271,711) [43].

### Strengths and limitations

There were some limitations of the study that should be considered. To ensure a balance between comprehensive data collection and participant burden, the post-intervention survey only asked about the community-facing intervention components and the best available measure of dose was the number of different sources of exposure. However, this is not the perfect measure and it is not expected that participants would be exposed to all the sources as some are aimed at specific segments of the audience. Additionally, because independent samples were used pre-and post-intervention, it was not possible to examine whether improvements in KAB or reduced salt intake were caused by different intervention exposures or dose received. The potential impact of having a project investigator undertake the interviews should also be acknowledged; however, it was agreed among the project team that she was the most culturally appropriate person to probe about how the context affected implementation and the mechanisms of impact, compared to a foreign interviewer. In addition, few food industry and community representatives participated in the interviews, and we were not able to interview to saturation, meaning other themes might be missed.

There were several strengths about this pragmatic national study of salt reduction interventions. It provides rare information about the challenges and facilitators of translating evidence-based research into real-world practice and policies from several perspectives including the government’s perspective as the program implementers. In contrast to the parallel project in Fiji, which was implemented by a research organization, this process evaluation helps understand the constraints and leverages that apply to governments—the main implementers of population salt reduction interventions [28]. Several sources of data and mixed methods were used so that information could be cross-checked for validity. The qualitative data were in broad agreement and provided rich context for the quantitative findings, which enhanced confidence in the findings.

## Conclusions

Given the complexity of population health programs, the process evaluation has been vital in holistically interpreting the study outcomes by accounting for the level of implementation and the context. MASIMA (in parallel with the partner project in Fiji [28]) was the first population-level study to include a process evaluation assessing the reach, dose, fidelity, cost, and context of a national, government-led salt reduction intervention in a lower-middle income setting. Based on these findings, we have formulated recommendations for future salt reduction interventions in Samoa and similar settings.

Future implementation of government-led salt reduction strategies in LMICs should comprise of individual and environmental-level interventions that account for contextual influences on food choices (e.g., price, availability, taste, convenience). Where possible, strategies should leverage or link to existing programs to increase feasibility and minimize cost. Easy-to-understand campaigns and behavior-change communication should initially educate consumers on the adverse health effects of salt consumption to improve motivation. More in-depth education (higher dose) should then transition to focus on providing practical advice and improving consumers’ capability to lower salt intake. In conjunction, policies or strategies that create environments that make lower salt consumption easier are needed. Although several high-income countries have successfully reduced salt intake through engaging food industry to voluntarily lower salt in foods, this may be challenging in LMICs with circumstances like Samoa. Legislative policies [5] should be considered in these settings as they create an even playing field for all food manufacturers and are theoretically more effective as it applies to all manufacturers as opposed to only those that have voluntarily committed [27]. More process evaluations of successful interventions are also needed to understand exactly how the interventions functioned within the context to achieve lower salt intake.

Population salt reduction is crucial for curbing the increasing prevalence of raised blood pressure in LMICs, [44] yet there is limited research on strategy implementation processes, costs, and effectiveness in these contexts. This study demonstrates that behavioral and community-based interventions alone, which had better reach and fidelity, are limited in effect without legislative backing in Samoa. Unlike high-income countries, initiatives to engage food manufacturers to voluntarily decrease the salt content of food products were less feasible to implement. Although it was challenging to implement legislation within the 18-month project duration, there is now increased consumer motivation and political readiness for legislative action. Continued long-term efforts to educate the population on practical ways to reduce salt intake and create environments that make eating lower salt more affordable, widely available, and appealing in terms of taste are needed to achieve a substantial and sustained reduction in salt intake in Samoa. It is hoped that the lessons learnt from the MASIMA process evaluation will also inform the development and implementation of effective salt reduction programs and policies in similar settings.

## Additional files

Additional file 1: Semi-structured interview quotes about contextual factors of implementation. (DOCX 21 kb)

Additional file 2: Semi-structured interview quotes about contextual factors affecting the intervention effects or mechanisms of impact. (DOCX 30 kb)

## Abbreviations

LMICs: Low- and middle-income countries
MASIMA: Monitoring and Action on Salt in Samoa
MESC: Ministry of Education, Sport and Culture
MoH: Ministry of Health Samoa
MRC: Medical Research Council
MWCSD: Ministry of Women, Community and Social Development
NCD: Non-communicable diseases
PEN FA’A SAMOA: Package of Essential Non-communicable Disease Interventions in Primary Care
PICs: Pacific Island Countries
WHO: World Health Organization

## References

[1] He FJ, Li J, Macgregor GA. Effect of longer term modest salt reduction on blood pressure: Cochrane systematic review and meta-analysis of randomised trials. BMJ. 2013;346:f1325. 10.1136/bmj.f1325 23558162

[2] Aburto NJ, Ziolkovska A, Hooper L, Elliott P, Cappuccio FP, Meerpohl JJ. Effect of lower sodium intake on health: systematic review and meta-analyses. BMJ. 2013;346:f1326. 10.1136/bmj.f1326 PMC4816261 23558163

[3] Mozaffarian D, Fahimi S, Singh GM, Micha R, Khatibzadeh S, Engell RE, Lim S, Danaei G, Ezzati M, Powles J (2014). Global sodium consumption and death from cardiovascular causes. N Engl J Med.

[4] Webb M, Fahimi S, Singh GM, Khatibzadeh S, Micha R, Powles J, Mozaffarian D. Cost effectiveness of a government supported policy strategy to decrease sodium intake: global analysis across 183 nations. BMJ. 2017;356:i6699. 10.1136/bmj.i6699 PMC5225236 28073749

[5] Trieu K, Neal B, Hawkes C, Dunford E, Campbell N, Rodriguez-Fernandez R, Legetic B, McLaren L, Barberio A, Webster J (2015). Salt reduction initiatives around the world - a systematic review of progress towards the global target. PLoS One.

[6] Mancia G, Oparil S, Whelton PK, McKee M, Dominiczak A, Luft FC, AlHabib K, Lanas F, Damasceno A, Prabhakaran D (2017). The technical report on sodium intake and cardiovascular disease in low- and middle-income countries by the joint working group of the World Heart Federation, the European Society of Hypertension and the European Public Health Association. Eur Heart J.

[7] Christoforou A, Trieu K, Land MA, Bolam B, Webster J (2016). State-level and community-level salt reduction initiatives: a systematic review of global programmes and their impact. J Epidemiol Community Health.

[8] McLaren L, Sumar N, Barberio AM, Trieu K, Lorenzetti DL, Tarasuk V, Webster J, Campbell NR (2016). Population-level interventions in government jurisdictions for dietary sodium reduction. Cochrane Database Syst Rev.

[9] World Health Organization (2007). Reducing salt intake in populations: report of a WHO forum and technical meeting, 5–7 October 2006, Paris, France.

[10] The World Health Organization (2010). Creating an enabling environment for population-based salt reduction strategies: report of a joint technical meeting held by WHO and the Food Standards Agency, United Kingdom, July 2010.

[11] Wyness LA, Butriss JL, Stanner SA (2012). Reducing the population’s sodium intake: the UK Food Standards Agency's salt reduction programme. Public Health Nutr.

[12] Karppanen H, Mervaala E (2006). Sodium intake and hypertension. Prog Cardiovasc Dis.

[13] TC HJE, Boon CS, Institute of Medicine (US) Committee on Strategies to Reduce Sodium Intake (2010). Strategies to Reduce Sodium Intake in the United States.

[14] Webster J, Snowdon W, Moodie M, Viali S, Schultz J, Bell C, Land MA, Downs S, Christoforou A, Dunford E (2014). Cost-effectiveness of reducing salt intake in the Pacific Islands: protocol for a before and after intervention study. BMC Public Health.

[15] Hou X, Anderson I, Burton-Mckenzie E (2016). Pacific possible: health and non-communicable diseases.

[16] Institute for Health Metrics and Evaluation (2017). GBD Compare.

[17] Trieu K, Ieremia M, Santos J, Neal B, Woodward M, Moodie M, Bell C, Snowdon W, Faumuina T, Webster J (2018). Effects of a nationwide strategy to reduce salt intake in Samoa. J Hypertens.

[18] Moore GF, Audrey S, Barker M, Bond L, Bonell C, Hardeman W, Moore L, O’Cathain A, Tinati T, Wight D (2015). Process evaluation of complex interventions: Medical Research Council guidance. BMJ.

[19] Glasgow RE, Vogt TM, Boles SM (1999). Evaluating the public health impact of health promotion interventions: the RE-AIM framework. Am J Public Health.

[20] Story M, Kaphingst KM, Robinson-O'Brien R, Glanz K (2008). Creating healthy food and eating environments: policy and environmental approaches. Annu Rev Public Health.

[21] Ministry of Health Samoa (2014). Salt Reduction Strategy and Action Plan 2014–2015.

[22] Linnan L, Steckler A (2002). Process evaluation for public health interventions and research: an overview.

[23] Liu H, Muhunthan J, Hayek A, Hackett M, Laba T-L, Peiris D, Jan S (2016). Examining the use of process evaluations of randomised controlled trials of complex interventions addressing chronic disease in primary health care—a systematic review protocol. Syst Rev.

[24] Samoa Bureau of Statistics (2017). Consumer Price Index - September 2017.

[25] Jick TD (1979). Mixing qualitative and quantitative methods: triangulation in action. Adm Sci Q.

[26] Centers for Disease Control and Prevention (2014). Best Practices for Comprehensive Tobacco Control Programs.

[27] Nghiem N, Blakely T, Cobiac LJ, Pearson AL, Wilson N (2015). Health and economic impacts of eight different dietary salt reduction interventions. PLoS One.

[28] Webster J, Pillay A, Suku A, Gohil P, Santos J, Schultz J, Wate J, Trieu K, Hope S, Snowdon W (2018). Process evaluation and costing of a multifaceted population-wide intervention to reduce salt consumption in Fiji. Nutrients.

[29] Trieu K, McMahon E, Santos JA, Bauman A, Jolly KA, Bolam B, Webster J (2017). Review of behaviour change interventions to reduce population salt intake. Int J Behav Nutr Phys Act.

[30] Michie S, van Stralen MM, West R (2011). The behaviour change wheel: a new method for characterising and designing behaviour change interventions. Implement Sci.

[31] Mozaffarian D, Afshin A, Benowitz NL, Bittner V, Daniels SR, Franch HA, Jacobs DR, Kraus WE, Kris-Etherton PM, Krummel DA, et al. Population approaches to improve diet, physical activity, and smoking habits: a scientific statement from the American Heart Association. Circulation. 2012;126(12):1514-1563. 10.1161/CIR.0b013e318260a20b PMC3881293 22907934

[32] Joint Standing Committee on Foreign Affairs Defence and Trade (2016). Food for thought: improving health and nutrition in the Indo-Pacific region.

[33] Owen KM. What do we know of consumers’ preferences and food choices in the islands of the South Pacific. In: Contributed paper to the 43rd Annual Agricultural and Resource Economics Society Conference. Christchurch; 1999.

[34] Mavoa HM, McCabe M (2008). Sociocultural factors relating to Tongans’ and indigenous Fijians’ patterns of eating, physical activity and body size. Asia Pac J Clin Nutr.

[35] Hardin J (2015). Everyday translation: health practitioners’ perspectives on obesity and metabolic disorders in Samoa. Crit Public Health.

[36] Drewnowski A, Specter S (2004). Poverty and obesity: the role of energy density and energy costs. Am J Clin Nutr.

[37] Seiden A, Hawley NL, Schulz D, Raifman S, McGarvey ST (2012). Long-term trends in food availability, food prices, and obesity in Samoa. Am J Hum Biol.

[38] Samoa Food Act 2015. 2015. http://www.palemene.ws/new/wp-content/uploads/01.Acts/Acts%202015/Food-Act-2015-Eng.pdf. Accessed 3 Oct 2017.

[39] World Health Organization (2016). Fiscal policies for diet and prevention of noncommunicable diseases: technical meeting report, 5–6 May 2015.

[40] Webster J, Trieu K, Dunford E, Hawkes C (2014). Target salt 2025: a global overview of national programs to encourage the food industry to reduce salt in foods. Nutrients.

[41] He FJ, Brinsden HC, MacGregor GA (2014). Salt reduction in the United Kingdom: a successful experiment in public health. J Hum Hypertens.

[42] Hope SF, Webster J, Trieu K, Pillay A, Ieremia M, Bell C, Snowdon W, Neal B, Moodie M (2017). A systematic review of economic evaluations of population-based sodium reduction interventions. PLoS One.

[43] The World Bank (2017). World Development Indicators.

[44] NCD Risk Factor Collaboration (NCD-RisC) (2017). Worldwide trends in blood pressure from 1975 to 2015: a pooled analysis of 1479 population-based measurement studies with 19.1 million participants. Lancet.

