# Supplementary material for: Process evaluation of Samoa’s national salt reduction strategy (MASIMA): what interventions can be successfully replicated in lower-income countries?
Source: Implement Sci. 2018 Aug 6;13:107. doi: 10.1186/s13012-018-0802-1 (PMC6080534; doi:10.1186/s13012-018-0802-1)
Supplement: Supplementary file 1 — Semi-structured interview quotes about contextual factors of implementation. (DOCX 21 kb) [file 13012_2018_802_MOESM1_ESM.docx]

Additional file 1. Semi-structured interview quotes about contextual factors of implementation

| **Theme** | **Respondent** | **Quote** |
| --- | --- | --- |
| Barrier of implementation of overall intervention: lack of staff | Related health or government org | No as I said before the barrier is lack of staff, the lack of ability to go out due to transport issues, due to other job responsibilities. |
|  | Related health or government org | Well staff is always a problem because we don’t have much staff. So what we do here in our office is we go out… They go out in the community weekly or fortnightly. Those are the only time they go out but mainly they are always stuck in the hospital. … It’s hardly known, this profession is hardly known in Samoa. Because of limited staff. Even in the hospital when they come in and told to see the dietitian, they don't know what's that. |
|  | MoH | Now for me working together with the salt team, I feel sorry for them because there’s only two of them and it’s a big project. I think that’s the only issue, shortage of the team. |
|  | MoH | So I had to pick up all those roles even though I didn’t understand how to do them but because of shortage of staff and just you know, the project needs to go on. …We could’ve done a lot better. Again it was only two of us |
|  | MoH | …so it was just them doing the work. But they managed to stay on board and continue the work |
| Barrier of implementing campaigns: high cost | MoH | It could have done better if we had a lot of money – it’s very costly here. …this was one area that we spent a lot of money on – the posters, the pamphlets, and all other resources that we had. …we could’ve outsourced it, but again it was trying to budget all that money that we had because it’s not cheap. |
|  | MoH | Unfortunately we didn’t have much money to print more. …Funding I think because we need a lot of money with interventions. … Promotion itself is very expensive |
|  | MoH | [When asked if there were any barriers] Financial. To have one ad in the TV, it’s expensive. And to have one page in the newspaper is expensive. |
|  | MoH | I just want for more funds for public awareness and community education. Because we need to have cooking demonstration that demonstrate the amount of salt to put in food. And then nutrition and planning for that. So I think we need more demonstration ideas. …Because of the financial issues. [In reference to why more mass media didn’t happen] |
|  | Related health or government org | The billboards very obvious – also this a question of location. …I do travel sometimes to the other island and to outside locations where I know they have other billboards. But I do know it’s expensive. |
|  | Related health or government org | this is the sexy thing with Samoa now, mass media which costs a lot. It’s very, very expensive and I can foresee it going up even higher. |
| Barrier of community mobilization of village women representatives: one-off session | MoH | Think what could’ve been improved is having ongoing sessions with them, not just when they get together but within the villages. Maybe we could’ve had monthly sessions from all around the island but again it’s hard to pull it all together. …[In relation to why that wasn’t done] Um because if we were to do that then it would basically mean it would be the whole year doing that. It’s a lot of villages. |
| Facilitator of community mobilization: existing mechanism to engage village women representatives | MoH | we’ve worked closely with the Ministry of Women who are basically the people who have direct access to the villages. So the programs that we have, we’ve worked with them and tried to take the message out to the villages. It’s not easy trying to go to all the villages so using this avenue was an opportunity, and it was a very successful one for us |
|  | MoH | With the Ministry of Women I think we got a lot with the program. There’s a program that’s basically increases basic knowledge of people regarding health, like basic hygiene and stuff like that. So we tacked into that and we talked to some of the reps from the Ministry of Women and we suggest to them the dangers of salt. For instance, especially in men, they have more hypertension so we talked to the reps from Women and explained to them the dangers of eating salt – high blood pressure, stroke, those kind of things. For them when they do the meetings, they present the dangers of that to village mayors with the hopes that they’ll go and spread the word. And that’s the same done with youth representatives and women representatives. So we basically talk to them and tell them why it’s dangerous to have too much salt. |
|  | Related health or government org | I think what your project, the Masima with the Ministry of Health, they went through the Division of Women and then they met once a month all these women committee members that come to Apia and then they have two hour sessions. And then other ministries can table agenda items. So that’s how your project was introduced. So they tabled the agenda this is the Masima project, they spoke to the women committee members, and that’s how they done the discussion back to the village. … Yeah definitely that’s very effective. In Samoa it’s very appropriate to do it like that. You cannot do it otherwise. …Of course, that’s the question because as you probably know the women committee members they are always the ones that… For instance the president is the wife of the high chief of the mayor. The secretary of the committee is the wife of the church minister. So all these committee members, maybe approximately ten to twelve all have very high hierarchies in the village. |
|  | Related health or government org | Right back to the villages. I think a lot of people are getting the message especially in the community through the Ministry of Women because I think that’s one of the most effective ways, through the Ministry of Women in the communities. Because those really work because they have programs within their own villages. And that’s where we get the message out to them. So I think a lot of people are getting the message about salt |
|  | MoH | Work together with some of the liaising officers, the village women representatives and the village mayor representatives, but they have that control over their village control and procedures. So we thought it’s a very good idea to have those controls so that they could push the people into as you say, behavioural change. We can’t change them over night, it takes time. But through those controls we have village council, we have our village women and mayor representatives, they have their own village by laws to push the people to like they have to comply with all these efforts that we are trying to convey. Especially the messages. … But within the controls coming from the leaders of the villages, those are the people that we have been working together. For us to push the messages and our technical advice and because they are the people who understand the behavior of the people that’s happening in the communities. |
|  | Community leader | I quite like the idea of engaging community leaders and because I mean the youth tend to look up to the decision makers. I think that’s really, really important. To be able to get your message out, I think the young ones need to be able to see the ones at the top actually implementing and doing it because there’s no point telling the young ones if you’re not doing it yourself. |
|  | MoH | Well most of the work that we do within the communities, we go through the Ministry of Women, and social development. …And I think that’s our gateway to the villages. …I think it’s easier to get it through the Ministry of Women because all they do is give us a list and at their monthly meetings we visit and say this is what we’re doing. I think it’s effective because if we go by ourselves, sometimes we can’t find them, sometimes we go there and they’re not available. But if we go through the monthly meetings and talk to them, present to them what it is and have a schedule and then go to the mayors |
|  | MoH | Yeah they have their roll out programs with salt. …And I think it works because that’s a general engine we use is through Ministry of Women. |
|  | Related health or government org | [In reference to the effectiveness of community engagement work with village women representatives] I think at the initial stage when we spoke with the team when we wanted to go out… I know there was a specific mention of targeting the women. Strategically of course you need to target the women because they’re doing most of the cooking. Not saying men don’t cook but in our culture women are also responsible/in charge for family obligations, child bearing and all of that. So having to work with the women and getting their level of understanding about the health implications or social implications through the project of course is a bonus. And that’s where the engagement with women is very important. |
| Barrier of implementing salt regulations: delay in the Food Act being passed | Related health or government org | No you cannot control it as you. This is you know a parliament process, things go slow and I know there has been a lot of lobby from the private sector to delay the food regulations. Nothing to do with salt, mainly on import and export issues with regards how they have to change how they package their food products. So they really try to influence members of parliament to delay it and yeah so that’s just politics. |
|  | MoH | It’s taken time because the draft regulations have been sitting there for three to four years. The delay was within parliament which was outside of our control. …But we don’t know why the delay – the Food Bill just got passed last June |
|  | MoH | we haven’t really gone into that side because we have also been waiting for our food bill to be passed which is passed now. It’s a Food Act last year. For the Food Act, we were hoping for that to back us up so that we can integrate the salt targets that we have received. …And they have also been mentioning the importance of waiting for the Food Act. I think it’s just unfortunate now the project is towards its end and now we have the Food Act– it looks like that’s the area we’re going to work on now which is why I’m hoping the Ministry will take up the project in this sense for what’s remaining and what we wanted to achieve. …As I said, we’ve been waiting for this Food Act because we’re also looking at labelling, an area we really need to push. … As I said, we’re just starting to do the ground work now. [in relation to the status of labelling work] |
| Barrier of engaging food industry to voluntary support salt reduction: waiting for Food Act | MoH | But in terms of trying to reduce salt, as I see it has been happening in Fiji, we haven’t really gone into that side because we have also been waiting for our food bill to be passed which is passed now … For the Food Act, we were hoping for that to back us up… but we have been working with the main ministries like Ministry of Industry Commence and Labour in terms of enforcement which is an area which we still need a lot of research to be done on but they are aware of what we are trying to achieve. And they have also been mentioning the importance of waiting for the Food Act. I think it’s just unfortunate now the project is towards its end and now we have the Food Act. …Basically the local producers. … We need to know, I mean basically, you want to not hurt them. Basically reach a mutual on how we can orbit together. |
|  | Related health or government org | Yeah I think they’re right [in relation to waiting for food regulations] because the industry here although it’s small, the ministry needs an environment where they can be in a leader position. Then they say okay this is the food regulation we have available – if you are accepting these regulations and want to work with us, this is the framework you follow. Because otherwise they will start, you know, not blaming them but maybe from the beginning they will say oh we are very committed but then they will start asking things from the ministry or like you know playing around with things like they supported for salt for other issues like soft drinks and you know there are a lot of small companies who are all the same families, so yeah I do understand the principle from the ministry. |
|  | MoH | At the same time considering that it’s a business … we’re really cautious because the Food Bill was not passed |

MoH- Ministry of Health, Org- Organization
