# Supplementary material for: Process evaluation of Samoa’s national salt reduction strategy (MASIMA): what interventions can be successfully replicated in lower-income countries?
Source: Implement Sci. 2018 Aug 6;13:107. doi: 10.1186/s13012-018-0802-1 (PMC6080534; doi:10.1186/s13012-018-0802-1)
Supplement: Supplementary file 2 — Semi-structured interview quotes about contextual factors affecting the intervention effects or mechanisms of impact. (DOCX 30 kb) [file 13012_2018_802_MOESM2_ESM.docx]

**Additional file 2: Table S2. Semi-structured interview quotes about contextual factors affecting the intervention effects or mechanisms of impact**

| Theme | Respondent | Quote |
| --- | --- | --- |
| Barrier to salt reduction amongst the public: salt and food is part of culture | Community leader | People love to eat here, and they love to eat salt. People love it so you got to make them not love it. … Everything is about food here. Everything. It’s huge what your job is. Because if you’re going to take that out you need to replace it because everyone here is living for the day. Everyone. Everyone here lives for today. Yeah we want a good future but you’re really living for the moment because not everyone has the food on their plate the next day. It’s more about we’ve got it here now let’s use it all, let’s use everything in our cupboard now. So you have to change the whole mentality not just about salt, but about eating. You’ve probably been to a big meal when all the food is out and… You take it and then you take another big plate home and eat it. So that’s the culture. So you’re dealing with a huge cultural issue. … But that’s honestly in this culture. At New Zealand when I’m home it’s like ‘stop that’s enough salt’ but over here it’s like ‘we just want to feed you, here take as much as you can’. To show that we love you, here have all the salt. |
|  | Related health or government org | We’ve been going out to the field and people love salt. And it’s habits and I think it’s a behavior that needs to change. … It’s always the attitudes. …No salt makes it tasteless. … when we have our meeting with the village women and we get served food. The salt comes out. And it comes out in a small bowl. You know, it’s not the table salt in a jar. It comes out in small saucer or small bowl. And then they just pass it around. And that’s the culture, that’s the attitude. “Oh it’s too tasteless, just add it in.” I think it’s just a shift in thinking again, just being a bit more conscious. |
|  | Related health or government org | I think it’s just their mindset because there’s the thinking that you only live once so you just enjoy whatever you enjoy. Some people that I’ve talked to, so when I say to them you have to reduce this and that. And they say we only have one life, just enjoy it as much as you can. … I think it’s just a mental like the mentality of people, they really need to work on. Yeah it takes a while. Because Samoans really used to their food and feasting. And trying to get through to them, it really takes a long time. |
|  | MoH | Some people they’re used to it. They’re very difficult to change to a new lifestyle. |
|  | Community leader | It’s the flavor I mean. I can’t be correct about this but I do know we love our food and so one is to have its flavor, second is to have quantity. The amount of the serving. And I think the most difficult aspect to it is that everywhere you go, every Samoan function, food is always involved. |
|  | Food Industry representatives | …people have got used to it and when it’s not there people notice it. The story of the three princesses really brings it home. … The father said to the three princesses give me what is most valuable to you. The first one gave gold, the second one gave diamonds and the third gave salt. And he said and that’s valuable, salt? And she said yes dad. Well I don’t think much of that. So she took all the salt out his meals the next day – by the end of the day, he was raving mad because his food was tasteless and he appreciated what she’d done. And that was something we were brought up on. And unfortunately I think you having to fight that story. … I think that people first of all you know, you go to cultural event – I call it funeral food, it’s all the stuff you’re not meant to have and there’s heaps of it. Very hard to say no and what can you do – you can’t throw it away so you eat it. It’s a problem. I don’t know the answer because it’s a mindset change that needs to occur. And the only time people have mindset change is when something quite debilitating happens in their life. |
|  | Food Industry representatives | I think it’s also just part of their tastes now. I think Samoans have noticed/grow up with a lot of salt being added to the diet. |
|  | Related health or government org | They used to those kind of food and they like eating it. |
|  | MoH | …the culture and traditions of how you usually eat foods… adding salt to food, especially meat and things is just so common. Like fish, they will add salt before eating. And pork. So that’s some behavior that needs to change. … So can’t just go and say cut down on the pea soup. That’s what they use at funerals and weddings |
|  | Community leader | I mean okay we love our food, it’s part of our culture but there should be a fine line drawing what’s healthy and what’s not. |
|  | Community leader | Samoan people love salt – for any feast or any meal, you have to add a lot of salt. We’re creating a lot of non-communicable diseases because of that. We have to change the attitude of our people in terms of eating habits. |
|  | Community leader | Well my knowledge about salt here in the small island of Samoa, salt is something that you need to add to your food. Without the salt, that means the food is not tasty. That’s my general knowledge for the food here in Samoa. Every food we eat, we cook, we have to add salt. I know Ministry of Health in Samoa is trying to do their program, especially last year, to let the people know that salt is very bad to their health. But to our general knowledge in Samoa, even me myself, if you are not adding salt to your food that means it does not taste good. … here in Samoa it’s very hard to change the old attitude and mentality of our old people. It’s very, very hard. |
|  | MoH | The other issue is trying to get through to them, changing their mindsets. Because they’ve been doing that, they’ve been living that life for so long. …People are being so used to eating salt that when something new comes in and trying to change their way of living, it takes… Well I say it takes courage for some people. It’s not easy |
| Barrier to salt reduction amongst the public: compared with unhealthy/salty food, healthy (low salt) food is more expensive, less convenient and less widely available | MoH | But sometimes the parents say the salt we can afford, we can’t afford the type of food you’re telling us to eat. We work, we don’t have time to go and plant it out in the back, which is understandable also |
|  | Related health or government org | And the types of food that we have to eat because sometimes I feel that’s available and what’s cheap is something that is convenient. Sometimes we eat salt with the Chinese meal. And people don’t understand. It’s the only available and cheapest that we’ve got access to |
|  | Related health or government org | All the food that we order [for hospital], we try to limit the salty food but we don’t know if we can maintain that, it depends on the money that we have. |
|  | Food Industry representatives | The tax structure we have is designed to make it cheaper for them to buy low quality food than to eat their own high quality food … Fruits and vegetables should be tax free. It’s not, it’s twenty percent. So when you look at it, the structure of our diet is determined by cost. We can’t afford very expensive stuff. …They’re good (in reference to the campaigns) but to get somebody when he comes up to Povi Masima (salted beef) at this price and rib eye is this price and you know the Povi masima is going to be more tender because its been soaked in chemicals and salt but you would choose the other one because it's cheaper. And that's what’s happening. It’s going to be hard to compete against. |
|  | Related health or government org | The other thing is those are the only foods which are affordable… It’s like the noodles. Yeah because that’s what we sell in our office everyday. …Yeah because it’s affordable, it’s easy to get. Not only here but at the canteen in the foyer, they all sell these kind of food. The other thing is the price. People go for the cheaper ones. Even though you know the awareness says it’s not good for health, but what else are we supposed to eat if we don’t have enough money – those kind of mindset and attitude. … But if the foods with salt are cheap like I mentioned before the Twisties, the noodles, people can’t stop eating them because they know they’re affordable. |
|  | Food Industry representatives | Another thing is, it’s always the cheaper option that’s most unhealthy. It’s what we can afford sometimes. Unlike countries overseas, the healthier options aren’t as pricey as us here in Samoa. The local produce is very low. They [fruit and vegetables] can be very expensive. Because of the change in weather and demand. It’s really hard for the crops to be consistent. Also consistency in taste like with us, we would go with the local option if it was you know consistent in quality but it’s not. |
|  | MoH | Yeah, the culture and traditions of how you usually eat foods and then just the competition with imported food coming in at low cost and just the accessible. Like you can easily go and get a meal from down the road that’s convenient. … So okay bread is high in salt, we don’t want to tell the public that. There needs to be a way to deliver it. Not so much worried about the culture but the concern is to deliver in a way because the foods that people normally consume maybe the ones that are high in salt and the ones they can only afford. |
|  | Community leader | I mean lots of the food that’s out in the rural areas, that’s the only food they can afford, so they can only buy those. And if the Ministry of Health despite their efforts in trying to promote ‘these are the foods that are high in salt, don’t eat these foods too much’, they would eat it because that’s all they can afford |
|  | Community leader | So most people just get the cheapest one even if they know it has a lot of salt. So basically they go for the cheaper food. |
| Barrier to salt reduction amongst the public: poor health and nutrition knowledge | Community leader | I guess we can start by educating our people – when you see people buying stuff, people shopping, people read about labels, does not know about the contents. When people buy tinned fish they think it’s safe but the more they have tinned fish, they’re having salt and all that. So when people buy they don’t have the information that allows them to be informed about what they’re doing. Some of our people do not have the privilege of understanding what that is. And so have that information and have people aware of the information and make decisions accordingly. …See I wouldn’t know that noodles have salt unless you said, I would have not known. … That’s another barrier to information, the language barrier. |
|  | Food Industry representatives | But that’s the other thing – education on top of that. The customers… I would guarantee it if you went downstairs and ask ten people if they think noodles has high salt or sodium, all ten would say not. The average consumer in the Pacific – not just Samoa – don’t know that noodles have high sodium. |
|  | MoH | So we also start to know from the community that they want to have more, they want to know but we were kind of afraid to teach them about the sodium reading the labels because of the level of health literacy. But now I see there’s different groups, don’t assume. Always be prepared with extra information if you need it. … We always had to be careful because if we get too technical, the information will be lost and the behavior – people will be like okay what do we actually do? |
|  | Community leader | I think it’s lack of education. …And it’s money in your pocket. And that’s what people actually care about, not your health because you don’t know what’s inside those packets, you don’t know what those numbers mean or anything. There’s a big lack of education about what’s right for you |
|  | Food Industry representatives | I think it’s all about increasing the people’s knowledge of what they’re eating. Sometimes you don’t do it because you don’t know about it. |
|  | MoH | So also the salt reduction messages information, you know we need to translate to simplest forms. Translation from English to Samoan is a challenge already – you need to find the right word. We do a lot of pre-testing sessions before it goes out and education sessions. Sometimes it’s the education sessions because WHO recommends 5g daily and then we have to make the equivalent. So that’s a teaspoon. Tell the people it’s a teaspoon like this. Or maybe we can find another way of measuring things easier for people. |
|  | Related health or government org | Yeah it takes a while. Because Samoans really used to their food and feasting. And trying to get through to them, it really takes a long time. But you just keep on knocking. And just explain it to them in really simple terms and they should understand. |
|  | Community leader | Sometimes the language is a bit of a barrier when we try to really thoroughly discuss an issue |
|  | Community leader | I liked it, they used very simple language and then they translated as well. …I mean not everyone is well, not everyone is educated and we tend to use very technical terms. |
|  | MoH | we’re not only doing it in English, we’re doing it in Samoan. And again, our focus in this area was to make the messages very simple for our people to understand. And we had to do pretesting and all that. |
|  | MoH | [When asked about any barriers] Language. Why I say language is because we always need to translate and some of the health vocabulary, it’s hard to find the right word for the health vocabulary and sometimes… Well we’ve been told by the prime minster, you people from Health Department, don’t have to use formal language when you deliver because people won’t understand you. … I think the biggest challenge is trying to get them to understand. … Because some people don’t understand what sodium is. |
| Facilitator of salt reduction amongst the public: increasing trend for healthier lifestyle in Samoa | MoH | So I think there’s more people wanting organic food now than from the past. And when I talk organic food in our context, I’m talking the Samoan food. Like the Samoan chicken is very different to the imported chicken because the chicken runs loose on land and it’s quite tough and that. I think people prefer the traditional diet now. |
|  | Community leader | Not fast food but cafes and healthy cafes. [In reference to more food places] Nourish and Cornwell, everyone’s got gluten free now. |
|  | Related health or government org | There’s a lot of fitness programs. A lot of people are conscious of what they eat now. … So I think there’s a change from ten years ago we love to feast whenever we have a chance. But now even when we order our food for events, we are really conscious, we really want to have healthier options. So I think it’s gradual. It’s taking time. |
|  | Food Industry representatives | But there’s also now, I think in the last year or so, for our customer base there’s been quite a significant move towards healthier eating. … A lot of them are going towards an organic sort of thing. There’s quite a few that are getting into a healthier lifestyle, and some are doing it because it’s quite popular so they are just jumping on board. But at the same time that’s creating quite a lot of moving towards that – so they’re checking the back of products, they’re wanting more products with less trans-fat, sodium, sugar. People are looking what’s in it like corn syrup, they don’t want it. |
|  | Food Industry representatives | And also the people have become very conscious now. …Yes I think it’s a trend that people are more conscious. …People are very serious in thinking about food. …I think it’s a timely project especially now that Samoans are very health conscious. |
|  | MoH | people are becoming more health conscious and they always try to do exercise… And some people mentioned in town, some vendors that sell like soda and things like that, they’re also starting to sell water. So… the message is out. |
|  | Community leader | I go to the gym…So I would come around 5 o’clock in the morning for classes. Before it was like only 2-4 people but now it’s like you have to be really early. Even lunch hour classes, before there were 5-7 of us but now a lot. I see a lot of people walking. I’m quite proud of how Ministry of Health has driven some of their initiatives, it’s really good. |
|  | Community leader | Most of our people in Samoa are starting to become health oriented in terms of eating habit, healthier lifestyle |
|  | Community leader | It’s business but there is a change but it’s not really a big change here in Samoa. There is new catering and new restaurants here in Samoa, they all cater for healthy food. It’s new over here, maybe last year or the last two years. It’s new here. Even at the moment, there are restaurants here that only cater for healthy food. And those healthy food, there’s no salt. So it’s coming up slowly. … A lot of people talking about healthy food now, especially fruit. |
|  | MoH | Some of the ads and the billboards have been effective in the sense that people are now starting to be health conscious, not just for salt, but for all the other health issues. |
| Barrier of community mobilization pathway of impact: limited knowledge | Related health or gov org | But they had barriers in the sense that they were asking ‘why are you asking me this?’. That’s when they told us we need more information, we want to give them a leaflet which explains this is what we are doing and this is our reason, we are helping. At first they try to explain from the knowledge they gained, but they don’t have that, so it would be very good if there’s ways to continue and see how we can provide them with information. |
| Barrier of community mobilization pathway of impact: saturation of messages | Related health or government org | I think it’s also not just about dissemination of messages. It has to be more than that, just like any other health message. …There needs to be another part of the package that goes into more to try and encourage behavior change. I’m trying to phrase this – the communities that we actually work in have been bombarded for years with lots of messages. |
| Facilitator of community mobilization effect: parallel PEN Fa’a Samoa NCD prevention program | MoH | According to the feedback that we have had from colleagues and WHO, when they went out to do the pilot, some of the villages requested salt reduction. They wanted to learn, know more about salt. And I think that was another way of knowing it was successful. …The PEN Fa’a Samoa was also a very useful event that this message was relayed. |
|  | Related health or government org | It stands for Package of Essential Non-communicable disease interventions in the Samoan way. And this project has a component of non-communicable disease awareness. And we ask villagers that participate in this project to set a project around salt, sugar, or tobacco. And actually a few of these villages actually have chosen salt and if we ask them why then I think this is attributed to the Masima project because they just saw these people are Women Committee members who have a role to go once a month to Apia. And they have been informed about this project through the meetings of the Ministry of Women… People are doing projects on salt and it’s very interesting. We ask them to write their own project proposal and so for instance one village every month when they have the council meeting, they monitor salt consumption. A little bit in their own way. The fact that they used to put salt in the middle of the meeting room. Because part of the meeting is to eat and salt is shared. But now they don’t do that anymore. So they hide the salt and then when the elderly ask I want the salt… They will give them information why the salt is not there, and they also have lemons to make changes. I think that’s very good. … In some other village, they do household visits. So they come in the house and they open the cupboard and they ask where is your salt and they ask when did you buy the new salt bag? … We have seven villages so I think there are four that chose salt. There’s quite a lot, I was quite surprised. |
|  | Related health or government org | So part of the community awareness the villagers were told to think of a project… And what do you know? Salt. And they were bold enough to talk about salts and requesting to do salts. …Now one of the villagers in Savaii said the reason why we want to do Masima is because we went to the community engagement that was done by the Ministry of Women, which was Merina. So the effect of going to other levels is awesome. So I think for the PEN Fa’a Samoa there was seven sub-villages and I think you have four… So that’s probably one of the things you can measure. Output of the Masima project. Also that it’s been continued by this PEN Fa’a Samoa. |
|  | MoH | And I think it works because that's a general engine we use is through Ministry of Women. And we’re not just doing salt, you do everything else along with it. I think with the PEN, I think that’s another mechanism that you can use to really get the salt message across. Because it’ll go with the food, the nutrition part. So I think it really needs to be hammered. And that’s the best because its village focused. |
| Barriers of food industry engagement impact: salty food sells and is more profitable | Food Industry representatives | They [Ministry of Health salt project officers] came down and spoke with me and I said my problem right now is salt sells, so does sugar. I really prefer Samoans to eat natural foods and to always eat what they traditionally ate. But unfortunately I’m competing against massive advertising campaign from the producers overseas here so I have to produce what the Samoans are eating. And that’s all there is to it. I don’t have a choice. If I don’t react to the market demand and I try to dictate to the market, I will lose. … They don’t need to convince me. I know I need to reduce. But I can’t change peoples’ choices and if I try to, I’ll lose my customers. I need my customers. |
|  | Food Industry representatives | At the moment we can’t sell, that’s our main concern – enforcing and how it’s going to be done and if it’s going to be an even playing field for everybody … Or whether it’s going to be lopsided – we’ll just go to these guys because they have proper documentation so we can follow up. The other guys might be in the ‘too hard’ basket so they won’t do that. |
|  | Food Industry representatives | We do try and use herbs you know when it comes to taste. Obviously salt makes things taste better but in substitute to that rather than using too much salt we add herbs and stuff to flavouring. Which is like actual flavouring. …I don’t think people really pick out that there’s more or less salt, just that it tastes good I guess… Over here it’s so hard for us because our people love the junk food. And if there’s hardly any salt in it, they say, there's no taste |
|  | MoH | Key challenges… Partnership with other industry. Concerns on cost and productivity. We have different targets, but you try to get a common ground on health, that’s most important. |
|  | MoH | Salt doesn’t cost much but they might be able to sell their product more if it’s saltier. |
|  | MoH | All they want is their money. … I think awareness programs through media campaign is important for those people. I think, if it’s hard, legislation will do the job, because once it becomes legislated they have to follow. But they’re money thinkers. They don’t worry about the content, all they want is to sell their goods. |
| Barrier of school nutrition standards: salty food sells and is more profitable | Community leader | You know whatever tastes nice sells and is more money in the pocket. …The thing is though, each school has their own person employed to provide the food. Each person, are not employed by the government or anything, they’re mainly self employed. So they come and they bring what they want. That’s the way it works. It doesn't work like its the schools funding, its her own funding. Its not about school as such because we tell her what to put in the canteen but if she doesn't want to make it, she's not going to make it - which is happening every term. Because it’s her business there’s only so much you can do. …It doesn’t work that way in Samoa [in relation to choosing another school food provider]. You often don’t release people because they don’t do what you tell them to do. Maybe in another country easy, but not here. |
| Barrier affecting food reformulation pathway to impact: salt used as a preservative | Food Industry | But preserving it in the heat is not actually that easy, salting tends to be natural preservative. So how to address that… Because refrigeration is very expensive. So you don’t have too much choice on what you can do. So I would say there’s quite a difficulty in addressing how we can reduce the salt intake because of the need for preservation and the heat to stop spoilage and also the fact alternatives are not that cheap. Salt and sugar are generally the cheap preservatives that you use for fruits, vegetables, and meat |
| Barrier affecting impact of school nutrition standards: caravans outside schools selling unhealthy foods | Related health or government org | I just did a few monitoring visits to the schools. I was a little bit disappointed in the sense that first of all the schools we visit, I would say they’re the better off schools. The school does a lot of effort to apply to the food and nutrition standards but then when you walk outside or even in the school compound, there is a little caravan. And that caravan is selling all the products that are not compliant with the nutrition standards. When we asked the principal they said they have a lot of difficulty because a lot of the schools are owned by the churches. So they have this board that reviews, the school board. So the church financially takes care of the school – school compound has caravan. So the money they gain from selling products around the school ground, is used for the school ground. So the school principal finds it very difficult to convince the church board to take out all these products. They said they did try, but the children don’t want that. I think there should be more efforts to setting up policy changes around the school environment. Like little shops around the school and working with local shop owners to make changes. |
|  | Related health or government org | Well that’s a long standing issue. You know those street vendors… And that’s a long standing issue. But the thing is, our mandates, our act, the education act, only regulated for school hours. After school hours then there’s no… And I think that’s the loop hole. … Yeah still vendors are still out during school hours. |
|  | MoH | There’s a lot of vendors outside school grounds and that is not allowed. And yet… they sell fizzy drinks, popcorn, salty foods, unhealthy stuff. Whereas within school nutrition standard, trying to promote healthy food – fruits and all that stuff. |
| Facilitator of school nutrition standards: high importance to improve children’s nutrition and their knowledge | Community leader | Yeah I think in terms of using school to ensure kids get the message out there. I think last year or the year before last, the Ministry of Education started to ban selling of salty foods inside the school compounds and trying to introduce the natural foods, the local ones. Things like paw paw. The healthier foods. Schools are getting used to that and some of the schools are still practicing that today. It’s good that they’re working in that sector environment rather than just the Ministry of Health and Education having different approaches. They’ve come together to make sure they have the same information. Especially for the kids, they are the future |
|  | Community leader | So my advice to… the Ministry of Health, for the next time you do this awareness, you have to starts from the schools, you have to bring the kids. You have to start from the schools. Because this old thinking with our old people, it’s very hard to change. So we have to educate our young ones. So the moment they grow up, they remember this. |
|  | MoH | The content of salt with the food and the result of that, having too much Masima especially for the school kids. Because now for the elderly, I think it’s too late for if they have diabetes, all result from this. But trying to get the message to the school kids in the early ages. |
|  | MoH | I think this is where we got to take a message especially our young population, this is the future of Samoa. The youth and the younger population. And we know that most of these preventive measures, it’s the children who really take the message home and start living by it rather than adults. Most children remind their parents that eating salt is not good. |
|  | MoH | I would include the schools. My personal feeling is that all the people, they’re getting old, they’re starting to go down. But if we go when they’re growing up, maybe we can save two or three lives if we go to them and educate their younger siblings and all the other kids in the schools. |
|  | Community leader | I have three children and I also teach them that. And they will ask why is it bad to eat salt and I will tell them. So now they will tend to very careful about certain things. I know as well if we start at a certain age, children can change. … And I think if we encourage and discuss those topics with our children at a very young age, they can follow. |
| Facilitator of salt reduction project and proposals for salt-related regulations: improved political readiness particularly around mandatory taxation of high salt foods | MoH | It’s good because the cabinet committee the salt progress report was presented and in this committee it’s all of the prime minister in cabinet, all ministries their CEO and their management. So our salt progress report was… And the minister of finance said you need to submit a paper and need to raise the taxes on salt which was a really good indication coming from the minister of finance of all people. So at least you know it’s there in the political scene because it’s them who decide. We just have to convince. |
|  | MoH | But later on, end of last year and this year, some of the information that was provided by the Masima project such as the amount of salt in different products… And in the regulations there’s a clause that says that just so the high salt/sugar/fat, we hopefully will have some policy options in the future that will have excised tax in future on foods which are high in those areas to help fund health promotion foundation. So that’s like a program that sort of from the salt, the information that is sort of being used to help with the food regulations as well as incorporating some of it into the nutrition program models that are in the food regulations. Like in terms of excised tax and reformulation because we know there’s a lot of high salt products in Samoa. |
|  | MoH | This [paper on salt project] is presented to the prime minister, cabinet, and all CEOs and aCEOS of the workforce. … We even make work easier when the minister of finance advises that put it all in paper and maybe increasing taxation foods high in salt can help reduce something. … Our finance was very well informed of when we did the presentation and he felt that by… According to the level of salt for each processed food, will be good. … As I said before we started this project most people don’t know the bad impact of salt. Most people are not conscious of the amount of salt they are taking in. Most people were not aware that taking too much salt can have a bad impact on your health. … And as I said today this was the comment made by the new minister of finance – start working on this now before the next budget. So we can propose more taxation on food high in salt, they now know salt is bad so we have to come up with a level where to start increasing the tax. …So they feel that by increasing taxation, people will slowly find other products – more healthier and cheaper. So I’m hoping that with the success of this salt project, now they said give it to me, we will push it… It happened because we just sent a paper to Cabinet and he was the only minister who commented and suggested that by putting a taxation. …And this is the way created by the salt project. |
|  | Related health or government org | Yeah taxation. The cabinet committee, the minister of finance was also keen to increase tax on salt. It’s going to be a good start on the selling point. … If it’s voiced by one of the minister who has control of the finances then I think to be it’s very good. Because it’s a high political decision making person. So I think I’m so proud of the minister who raised that. |

MoH- Ministry of Health, Org- Organization
